# Rapid and sensitive RPA-Cas12a-fluorescence assay for point-of-care detection of African swine fever virus

**DOI:** 10.1371/journal.pone.0254815

**Published:** 2021-07-19

**Authors:** Jinyu Fu, Yueping Zhang, Guang Cai, Geng Meng, Shuobo Shi

**Affiliations:** 1 Beijing Advanced Innovation Center for Soft Matter Science and Engineering, College of Life Science and Technology, Beijing University of Chemical Technology, Beijing, China; 2 College of Veterinary Medicine, China Agricultural University, Beijing, China; University of Nicolaus Copernicus in Torun, POLAND

## Abstract

African swine fever (ASF) is a serious contagious disease that causes fatal haemorrhagic fever in domestic and wild pigs, with high morbidity. It has caused devastating damage to the swine industry worldwide, necessitating the focus of attention on detection of the ASF pathogen, the African swine fever virus (ASFV). In order to overcome the disadvantages of conventional diagnostic methods (e.g. time-consuming, demanding and unintuitive), quick detection tools with higher sensitivity need to be explored. In this study, based on the conserved *p72* gene sequence of ASFV, we combined the Cas12a-based assay with recombinase polymerase amplification (RPA) and a fluorophore-quencher (FQ)-labeled reporter assay for rapid and visible detection. Five crRNAs designed for Cas12a-based assay showed specificity with remarkable fluorescence intensity under visual inspection. Within 20 minutes, with an initial concentration of two copies of DNA, the assay can produce significant differences between experimental and negative groups, indicating the high sensitivity and rapidity of the method. Overall, the developed RPA-Cas12a-fluorescence assay provides a fast and visible tool for point-of-care ASFV detection with high sensitivity and specificity, which can be rapidly performed on-site under isothermal conditions, promising better control and prevention of ASF.

## Introduction

African swine fever (ASF) is an epidemic disease that endangers both domestic and wild pigs. Since its first identification in Kenya in the 1920s, it has spread quickly in Africa and Eurasia [1]. Highly contagious and deadly, this infectious disease has caused devastating economic damages to the swine industry, with risks in the place where ASF epidemics occurred. The first recent case of ASF was reported in China in 2018, in Liaoning province, followed by an outbreak of ASF across the country, which has led to the death of tens of thousands of pigs to date [2]. African swine fever virus (ASFV) is the pathogen of ASF, a double-strand DNA virus with a complex structure, transmitted by arthropods- in this case, soft ticks-of the *Ornithodoros* genus.

This indicates that direct contact and ingestion of contaminated materials can lead to virus transmission and spread. Based on the C-terminal region variation of the gene (B646L) sequence encoding the major capsid protein p72, 24 different genotypes have been classified [3,4]. According to the severity of ASF, the clinical signs could be divided into three types: acute, subacute and chronic [5]. Although the ASF virus has no interrelationship with the classical swine fever (CSF) virus, differences between CSF and ASF are indistinguishable in both clinical signs and postmortem autopsy results [6].

Strict hygiene measures and effective diagnostic methods are critical to prevent inter-country transmission when there is neither an effective vaccine nor treatment. The World Organization for Animal Health (OIE) has recommended several diagnostic methods for ASFV, including fluorescent antibody tests (FAT) of the antigen, or polymerase chain reaction (PCR) of the virus genome [7]. Even though this gold standard has been accepted for years, early infection cannot be detected, limiting wide application. Prominent molecular tools, such as quantitative real-time polymerase chain reaction (qPCR), are known for their sensitivity and specificity; however, their high time consumption, along with complex equipment and professional operating requirements, make qPCR unconducive to on-site detection. Other diagnostic methods are in the process of development for better detection of ASFV as quickly as possible.

Recently, clustered regularly interspaced short palindromic repeats (CRISPR) and CRISPR associated (Cas) protein have been prominent in gene editing and nucleic acids detection. Based on the adaptive immune system found in prokaryotes (bacteria and archaea), which helped in targeting and degrading the foreign nucleic acids (i.e. plasmid and phage), the CRISPR/Cas system led to a notable advancement in biotechnology [8]. Alongside the Cas9 protein, which was utilized in gene editing in living cells for several years [9], Cas12a (previously called Cpf1) has recently been shown to be a promising prospect for nucleic acid detection with the advantages of high specificity and sensitivity. Detection relies on the ribonucleoprotein complex, which takes over the function of proto-spacer-adjacent motifs (PAM) recognition. The specific hybrid between crRNA and target dsDNA activates indiscriminate ssDNA sever, displayed by Cas12a [10]. Using a fluorophore quencher (F-Q)-labeled reporter assay, identification can be visible under blue light with the naked eye. Combined with isothermal amplification, Chen *et al*. designed a method termed DNA endonuclease-targeted CRISPR trans reporter (DTECTER), which enabled sensitive and specific detection, as well as the genotyping of the human papillomavirus in both cell lines and clinical samples [11].

Latterly, CRISPR based technology has taken giant steps in biomedical diagnosis. For example, Gootenberg *et al*. created a CRISPR-Cas13a-based diagnostic platform called SHERLOCK (specific high-sensitivity enzymatic reporter unlocking) with a combination of RPA and a fluorophore reporter, which achieved detection of specific strains of Zika virus (ZIKA) and Dengue virus (DENV) from body fluids [12]. CRISPR-Cas12a has already been applied in practice, especially in contagious pathogen detection, such as COVID-2019 [13,14], SARS-CoV-2 [15,16] and ASFV [17–19]. It is expected that CRISPR-based technology will become a promising diagnostic tool for future pathogen detection.

**Fig 1:**
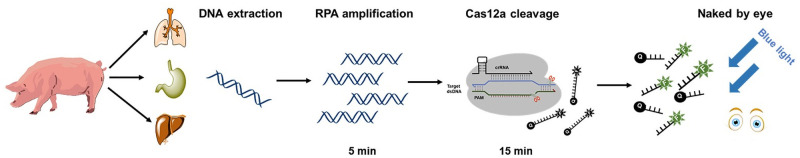
Schematic of the CRISPR-Cas12a based assay for ASFV detection. Steps are summarized as follows: (1) Extraction of the DNA from pig tissues; (2) Amplification of the target using RPA method; (3) Cas12a cleavage; (4) Fluorescence signals detected by the naked eye.

As described in this paper, we developed an RPA-Cas12a-fluorescence assay targeting the *p72* gene for the detection of ASFV (Fig 1). This method amplifies the *p72* gene using RPA within five minutes, and can complete the Cas12a cleavage assay within 15 minutes. Rapid and sensitive, it can be undertaken without complicated equipment, and is easier to use for point-of-care diagnosis in the fieldwork for vets.

## Materials and methods

### Generation of dsDNA targets

PCRs were performed by Q5 High-Fidelity DNA Polymerase (New England Biolabs, Inc, USA) with the primers (Table 1) and 2 μL genome samples, following the program: 98°C for 30 seconds, then 35 cycles of 98°C for 5 seconds, 58°C for 10 seconds and 72°C for 30 seconds. Primers for RPA are listed in Table 1. RPA reactions were performed by the Twist-Amp basic kit (TwistDX, British). Each RPA reaction contained 29.5 μL rehydration buffer, 2.4 μL forward and reverse primers, 2 μL genome samples, 2.5 μL of magnesium acetate (MgAc), and 11.2 μL water. The mixtures were incubated at 39°C for 20 minutes, then the RPA products were cleaned using the alcohol precipitation method and verified by electrophoresis on a 1% agarose gel.

**Table 1:** Primers and crRNA used in this study.

Method	Name	Sequences (5’-3’)
**PCR**	p72-F	TTAGGTACTGTAACGCAGCACAGCTGAAC
	p72-R	ATGGCATCAGGAGGAGCTTTTTGTCT
**RPA**	p72-RPA-F	CAACTTAATCCAGAGCGCAAGAGGGGGCTGATAG
	p72-RPA-R	TGGAGGGAACCAGTGGCCCTCTCCTATGCAAC
**crRNA**	crRNA-R	GAAATTAATACGACTCACTATAGGG
	crRNA-1-F	GGTTGGTATTCCTCCCGTGGCTTATCTACAACAGTAGAAATTCCCTATAGTGAGTCGTATTAATTTC
	crRNA-2-F	TCAATAACCTGTTTGTAACCCCTATCTACAACAGTAGAAATTCCCTATAGTGAGTCGTATTAATTTC
	crRNA-3-F	TCTCACAATATCCAAACAGCAGGATCTACAACAGTAGAAATTCCCTATAGTGAGTCGTATTAATTTC
	crRNA-4-F	AACCCACTTTGAGTCAAATCGAAATCTACAACAGTAGAAATTCCCTATAGTGAGTCGTATTAATTTC
	crRNA-5-F	CTGAATAGCAGGATCTCTAACATATCTACAACAGTAGAAATTCCCTATAGTGAGTCGTATTAATTTC

### Cas12a expression and purification

A his-tagged (C-terminal) codon-optimized version of Cas12a (*Francisella tularensis*) gene was ordered from Sangon Biotech (Shanghai, China). The expression plasmid (FnCas12a- pET28a) was transformed into BL21(DE3), then BL21(DE3) cells carrying the expression plasmid were cultured in Luria-Bertani (LB) medium at 37°C overnight. The cells were transferred into fresh LB (1:100 inoculation) at 37°C until OD600 reached 0.8, then induced with 0.5 mM IPTG and expressed at 18°C for 16 hours. Cells were collected by centrifugation and resuspended in 50 mL of lysis buffer [50 mM Tris-HCl (pH 8.0), 1.5 M NaCl, 1 mM DTT and 5% glycerol] with 1 mM phenylmethanesulfonyl fluoride (PMSF) as the protease inhibitor and lysed using high pressure. After centrifuged at 15,000 g for 30 minutes, the collected supernatant was loaded onto the HisTrap HP column (GE Healthcare, USA). The column was then washed with wash buffer (lysis buffer supplemented with 30 mM imidazole) and eluted with elution buffer (lysis buffer supplemented with 600 mM imidazole). The collected protein was dialyzed in a storage buffer (20 mM Tris-HCl, pH 8.0, 600 mM NaCl, 1 mM DTT, 0.2 mM EDTA, 15% (v/v) glycerol) and finally stored in aliquots at -80°C.

### Transcription of crRNAs

crRNA preparation proceeded in three steps. The transcription templates for crRNA preparation were amplified by the PCR process. Primers are listed in Table 1. Then, the transcription process was performed at 37°C overnight, using the T7 High Yield Transcription Kit (Thermo Fisher Scientific, USA). Finally, the transcript products were purified using the RNA Clean & ConcentratorTM-5 (Zymo Research, USA) and quantified with Nano-Drop 2000c (Thermo Fisher Scientific, USA).

### Cas12a detection

The Cas12a cleavage reaction system contained 500 nM Cas12a, 500 nM crRNA, 2 μL target DNA, 500 nM ssDNA (HEX- GATCAAGAGCTA -BHQ1) and 0.5 μL RNase inhibitor (TaKaRa, Japan) in a 20 μL volume. The reactions were performed at 37°C in NEB buffer 3.1 for 15 minutes, and the fluorescence signals were examined using EnSpire (PerkinElmer, USA).

### Visual detection

The ssDNA reporter was replaced in the Cas12a reaction with ssDNA reporter (FAM- GATCAAGAGCTA -BHQ1), and then Azure C300 Gel Imager (Azure Biosystems, USA) was used to examine the fluorescence signals by the naked eye under blue light.

### Specificity and sensitivity of Cas12a-based ASFV fluorescence reporting system

The sensitivity of the Cas12a-based assay was determined with a concentration gradient of target dsDNA, adjusted to be 0.05nM, 0.1nM, 0.15nM, 0.2nM, 0.25nM, 0.5nM, 1nM, 1.5nM, 2nM, 2.5nM and 5nM. Every reaction process was replicated three times.

### Sensitivity of RPA-Cas12a-fluorescence assay

With a copy number gradient of 2×10^0^, 2×10^1^, 2×10^2^, 2×10^3^, 2×10^4^, 2×10^5^, 2×10^6^, 2×10^7^ and 2×10^8^, the dsDNA target was engaged respectively in the RPA reaction. Visualization of the RPA product was detected by agarose gel electrophoresis. The Cas12a-based assay was performed with the above RPA products. Meanwhile, different reaction times of the Cas12a-based assay were determined with 5 minutes, 10 minutes, 15 minutes, 20 minutes, 25 minutes and 30 minutes. Each reaction process was replicated three times.

### Ethics statement

No ethics statement is required for this work. Nucleic acids of ASFV were not isolated in this work, which was a gift sample from the Animal Disease Prevention and Control Center of Liaoning province [20].

## Results

### Design of crRNA guides and RPA primers

**Fig 2:**
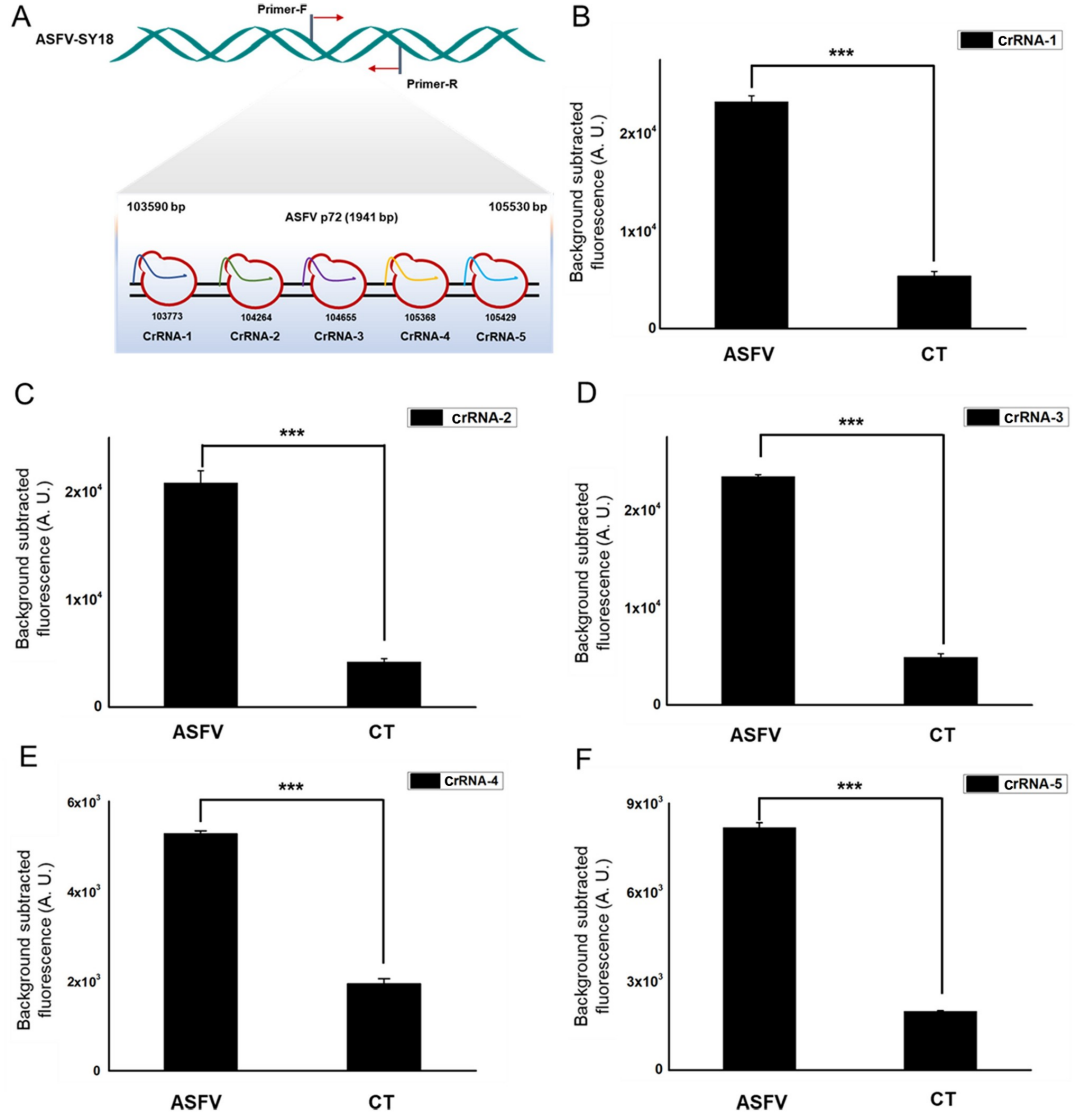
Screening for highly active crRNAs. (A). Schematic of the *p72* gene, and the corresponding locations of each crRNA PAM region. (B-F). Fluorescence signals detected by five crRNAs targeting the ASFV *p72* gene designed for the detection of ASFV. Background fluorescence by the individual crRNA in the absence of target DNA was shown as “CT”. n = three technical replicates, bars represent mean ± SD, ***p <0.001.

The *p72* gene, considering its conservative property, was chosen as the target sequence for crRNA design. Based on the sequence of *p72* gene of ASFV found in the ASFVdb and GenBank, a pair of primers (p72-RPA-F and p72-RPA-R) used in RPA reaction was designed for the target dsDNA enrichment. It was crucial that the appropriate crRNA had high specificity, to specifically pair with the target strand of dsDNA [9]. In our study, five specific crRNAs were designed and prepared by *in vitro* transcription (Table 1) (Fig 2A).

*In vitro* study showed the cleavage results of five specific crRNA guides. The use of crRNA-1, crRNA-2, crRNA-3, crRNA-4 and crRNA-5 in the Cas12a-based assay had a significant statistical difference (p<0.001, student’s T-test) in fluorescence intensity compared with the negative group. The results indicated that all five crRNA we designed can guide the Cas12a cleavage activity with remarkable specificity (Fig 2B–2E).

### The sensitivity of Cas12a-based ASFV fluorescence reporting system

**Fig 3:**
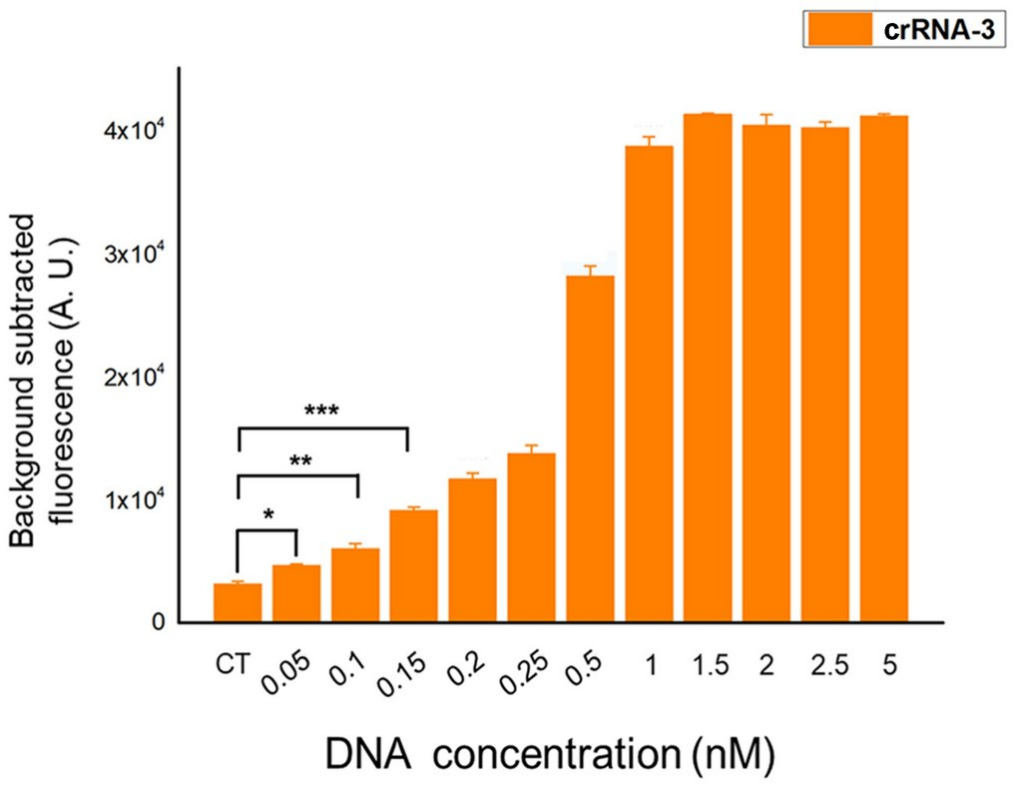
Sensitivity of the Cas12a-based ASFV fluorescence reporting system. Cas12a assay can detect as low as 0.05 nM of PCR products. n = three technical replicates, bars represent mean ± SD, ***p <0.001, **p<0.01, *p<0.05.

Next, we selected crRNA3, which generated the greatest fluorescence signal to detect the limit of the target DNA. With the target dsDNA (PCR production) concentration gradient of 0.05nM, 0.1nM, 0.15nM, 0.2nM, 0.25nM, 0.5nM, 1nM, 1.5nM, 2nM, 2.5nM and 5nM, the sensitivity of the Cas12a-based assay was verified. Background fluorescence of the individual crRNA in the absence of target DNA was defined as the negative group (CT). The results showed that even though it did not reach a significant value (p<0.001, student’s T-test) found a DNA concentration of 0.05nM between the experimental group and the negative group, but a difference could be identified (p<0.05, student’s T-test). With a DNA concentration of 0.1nM, the statistical analysis showed the enhanced fluorescence intensity (p<0.01, student’s T-test) in response to the negative group. Until the DNA concentration reached 0.15nM and above, the results showed a significantly higher difference (p<0.001, student’s T-test) compared with the negative group (Fig 3). Thus, the results indicated that the Cas12a-based ASFV fluorescence reporting system could detect as low as 0.05nM of DNA concentration.

### The sensitivity of RPA-Cas12a-fluorescence assay

**Fig 4:**
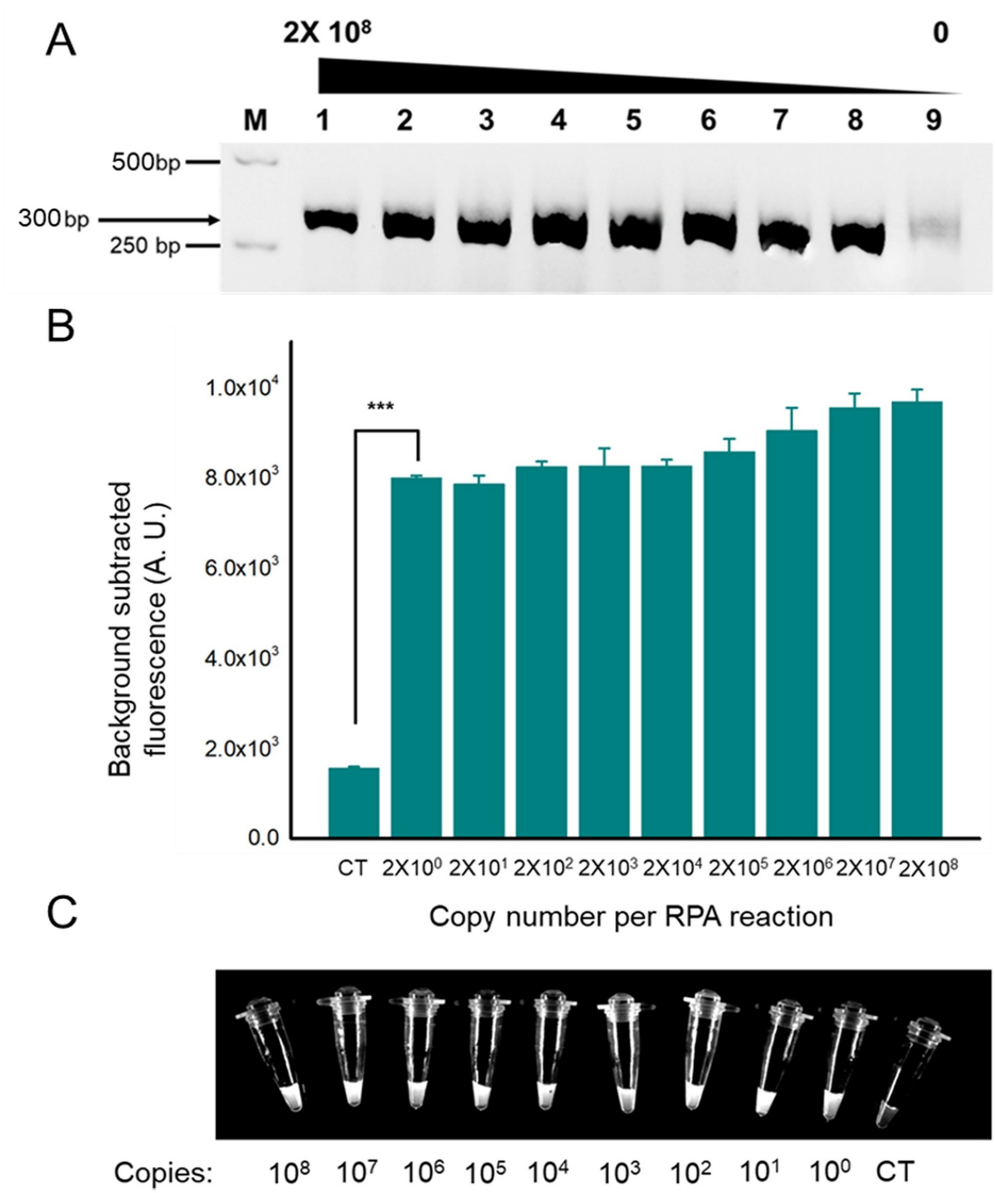
Detection of ASFV using RPA coupled with Cas12a-based assay. (A). Visualization of RPA products by agarose gel electrophoresis. The expected product size is 300 bp. (B). Reactions were performed after an RPA amplification of the virus DNA with varying initial concentrations, and it can detect as low as two copies of initial DNA. n = three technical replicates, bars represent mean ± SD, ***p <0.001. (C). Direct observation by the naked eye when the Cas12a reactions system is exposed to blue light. CT, negative control.

We established the RPA-Cas12a-fluorescence assay based on the Cas12a cleavage activity combined with RPA reaction. RPA was used to amplify the sample DNA, and RPA products were verified by agarose gel electrophoresis. Under UV light, a concentration gradient of sample DNA can produce remarkable bands of 300bp in every lane. Meanwhile, the negative group showed no bands (Fig 4A).

After the RPA reaction, fluorescence detection was performed with the participation of a fluorophore-quencher (FQ)-labeled reporter. The sensitivity of this assay was confirmed by using different target DNA copy numbers of RPA reaction. After fluorescence signal acquisition of reactions with a copy number gradient of 2×10^0^, 2×10^1^, 2×10^2^, 2×10^3^, 2×10^4^, 2×10^5^, 2×10^6^, 2×10^7^ and 2×10^8^, the statistical analysis revealed that there were significant differences in fluorescence intensity (p<0.001, student’s T-test) between all experimental groups and negative groups (Fig 4B). Under blue light, every tube appeared to have remarkable fluorescence, distinguishing them from the negative group (Fig 4C). Thus, Cas12a assay can detect as low as two copies of the genome and observed with naked eyes.

**Fig 5:**
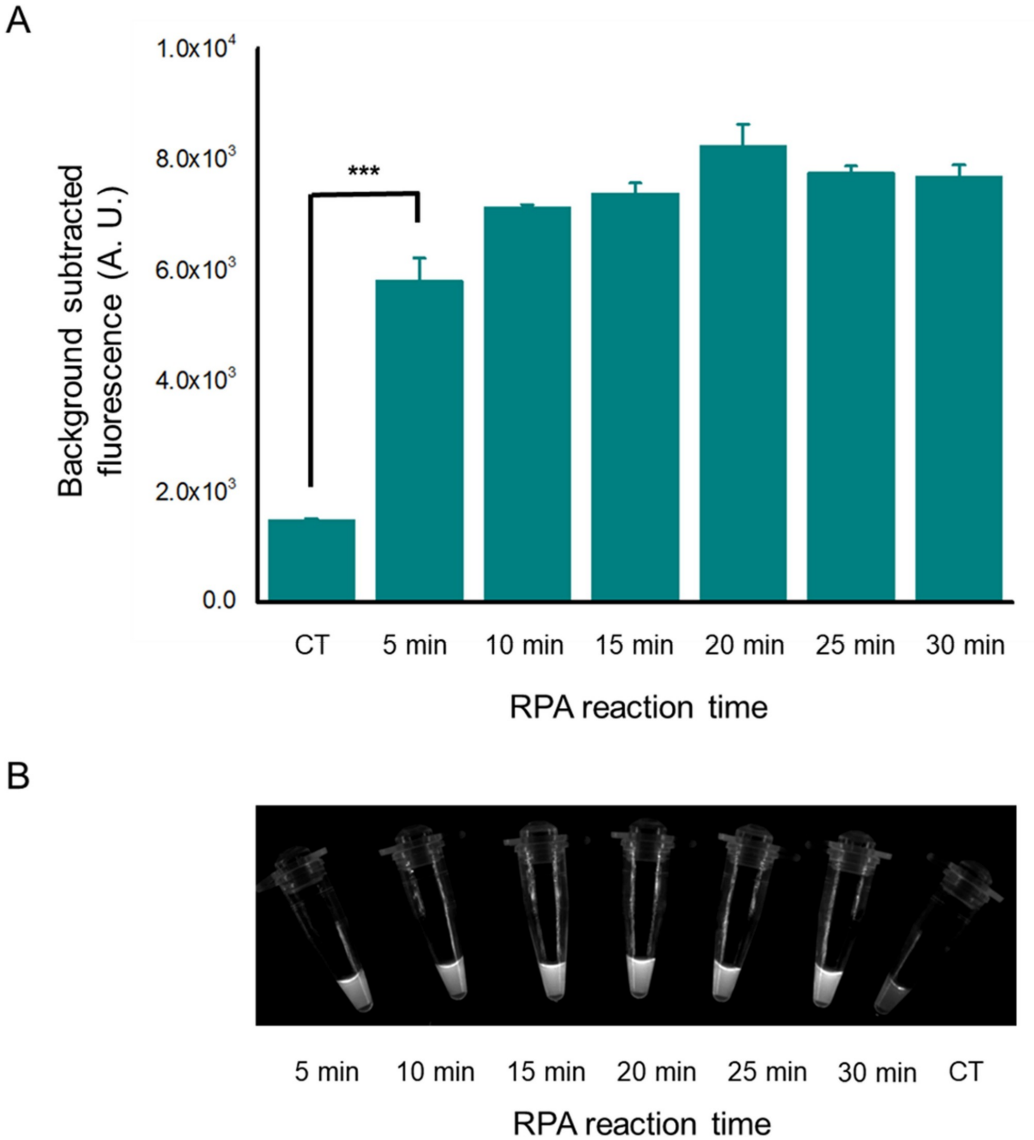
The detection result of different RPA reaction times. (A). Florescence signal when RPA was performed at varying times from 5 to 30 minutes. The RPA amplified product reacted for 5 minutes can be detected by Cas12a-based assay. The template of RPA is two copies of the genome. n = three technical replicates, bars represent mean ± SD, ***p <0.001. (B). Direct observation by the naked eye when the Cas12a reactions system are exposed to blue light. The detected nucleic acid samples were obtained by RPA at varying time from 5 to 30 minutes from 2 copies genome. CT, negative control.

To determine the appropriate reaction time of RPA, we used two copies of the genome as a template to perform RPA at varying times from 5minutes to 30 minutes. All groups showed that the Cas12a-based assay can produce significant fluorescence intensity (p<0.001), different from the negative group (Fig 5A). Under visual inspection of the corresponding reactions from Fig 5A, the distinct results were showed and confirmed that RPA-Cas12a-based assay can detect two copies of viral DNA (Fig 5B). As the RPA takes a minimum time of 5 minutes and the Cas12a-based assay needs 15 minutes, the total reaction time could be as short as 20 minutes when the viral DNA was extracted.

## Discussion

The grim situation of the pandemic disease African swine fever (ASF) has attracted world attention in the last few years. ASF is a highly contagious viral disease in domestic and wild pigs, leading to lethal hemorrhagic fever with high mortality of nearly 100% [3]. Because of the relatively short period from symptom onset to outcome [18] (within 6–13 days or up to 20 days of the acute form [7]) and the distant outbreak sites from diagnostic laboratories, shorter diagnostic time and easy manipulation in the field are eagerly demanded. Given that diagnosis and treatment will lead to greater exposure and more severe contagion, a rapid, point-of-care detection method was required for timely intervention to control and prevent wide transmission.

Recently, a variety of laboratory tools has emerged to solve the immediate problems for better detection of ASFV, such as serodiagnosis (immunoelectroosmophoresis, IEOP) test [21], antigen ELISA [22], fluorescent antibody test (FAT) [23], immunochromatography test strip (ICTS) [24]) and molecular tools (PCR, RPA [25], multiplex RT-PCR [26] and qPCR [27,28]). The OIE has recommended several ASFV detection methods, including virus isolation, fluorescence antibody test (FAT) and conventional PCR or real-time PCR [29] in 2018. As reliable indicators of infection, antibodies used to be the consistent and most economical method for ASFV detection. However, in some cases, such as when infected animals died prior to the occurrence of antibodies, serodiagnosis was unable to be used for diagnosis [30].

Real-time PCR as an effective molecular tool had the desired effect for ASFV detection. Nevertheless, real-time PCR requires highly complex equipment and qualified personnel. At the same time, the clinical samples need to be transported to a laboratory for completing the test and obtaining the final results. These conditions impose restrictions on real-time PCR application on point-of-care detection in the field. Recently, several laboratory studies created detection method based on CRISPR technology and lateral flow strip detection [17–19], which is semi-quantitive and not particularly sensitive (at least 20 copies of the genome can be detected). Thus, the development of a simple and rapid ASFV early infection detection test was demanded to overcome time and operational problems.

We designed a CRISPR-Cas12a based assay combined with RPA reaction for ASFV detection (Fig 1). Clinical samples were obtained from the farms and then nucleic acids were extracted from pig tissues on-site. Initially, the target dsDNA was amplified using the RPA method for product enrichment; the Cas12a based cleavage assay was performed, collecting fluorescence signals. The diagnostic results can be recognized by naked eye or fluorescence analysis. With the above processes, we can achieve a rapid and simple assay for point-of-care detection of ASFV.

In our study, five specific crRNAs were designed for precise detection. Every crRNA in this experiment showed remarkable specificity in target dsDNA recognition. In the CRISPR-Cas12a cleavage activity efficiency test, the Cas12a expressed and purified in our laboratory proceeded well with as little as 0.15nM DNA showing a significant effect on target detection.

To examine the RPA-Cas12a-fluorescence assay efficiency, we found that reactions can be detected with as few as two copies of the genome. Meanwhile, the whole Cas12a based cleavage assay can detect RPA products in just 5 minutes. All the above indicated that the assay we designed has great sensitivity, specificity, and high efficiency.

In this experiment, we have not applied our assay in clinical samples (due to biosafety regulations), but it worked very well in the lab experiment. We now require greater variety of samples, such as anticoagulant blood, spleen, tonsils, lymph nodes, kidneys or bone marrow in order to perform a further evaluation of different samples in the future.

In summary, we developed an RPA-Cas12a-fluorescence assay for rapid detection of the ASFV genome, which can be performed without complex equipment and with the ability to be processed in only 30–40 minutes. The test results can be obtained under visual inspection and fluorescence intensity analysis. It promises to be a potential application for point-of-care diagnosis, which can be used on-site in field research.

## Supporting information

S1 Raw images (PDF)

S1 Data Original data for Figs 2–5. (XLSX)
