# Supplementary material for: Rapid and sensitive RPA-Cas12a-fluorescence assay for point-of-care detection of African swine fever virus
Source: PLoS One. 2021 Jul 19;16(7):e0254815. doi: 10.1371/journal.pone.0254815 (PMC8289064; doi:10.1371/journal.pone.0254815)
Supplement: S1 Raw images — (PDF) [file pone.0254815.s001.pdf]

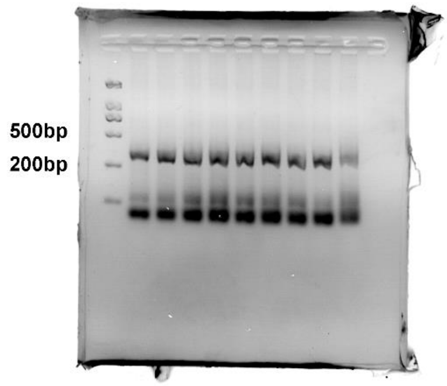

Figure 4A was generated from this original image.

The RPA amplification result of the virus DNA with varied initial concentrations. From the left to the right concentration respectively is 2×10^8, 2×10^7, 2×10^6, 2×10^5, 2×10^4, 2×10^3, 2×10^2, 2×10^1, 2×10^0 copies/μL.

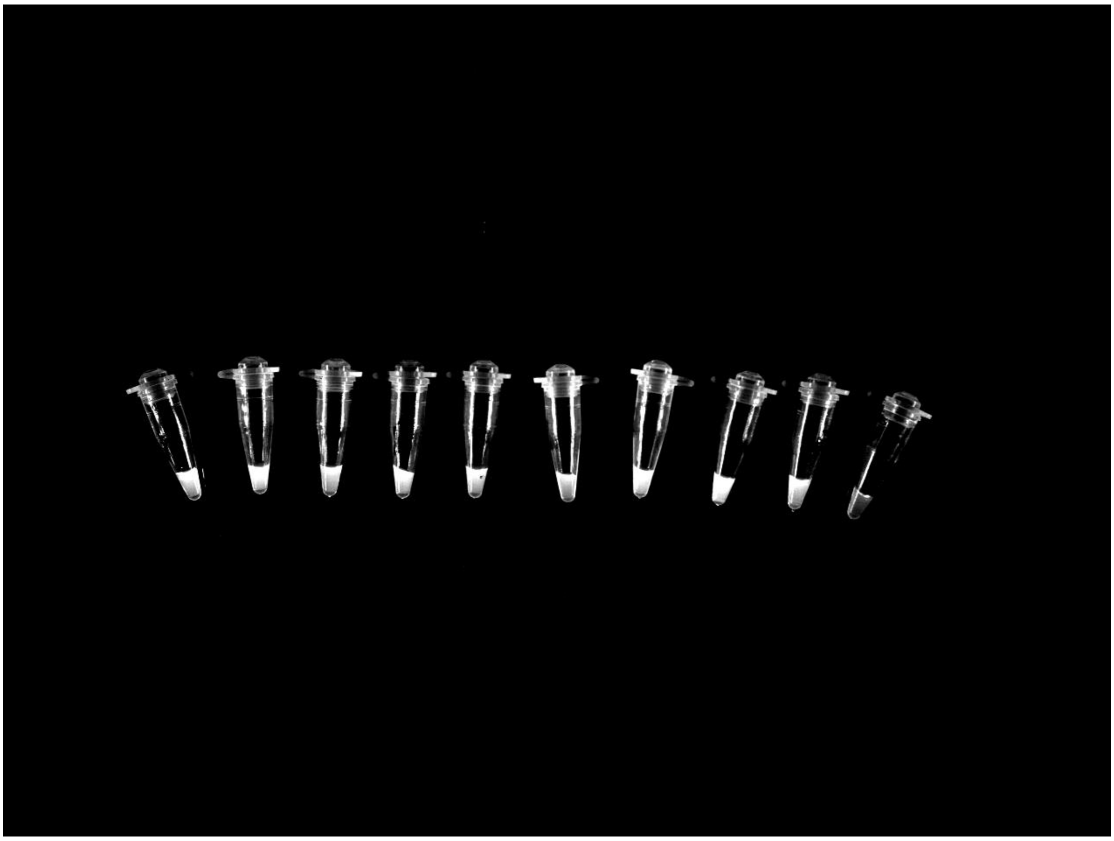

Figure 4C was generated from that original image.

Direct observation by naked eye when the Cas12a reactions system exposed to blue light. From the left to the right concentration respectively is 2×10^8, 2×10^7, 2×10^6, 2×10^5, 2×10^4, 2×10^3, 2×10^2, 2×10^1, 2×10^0 copies/μL.

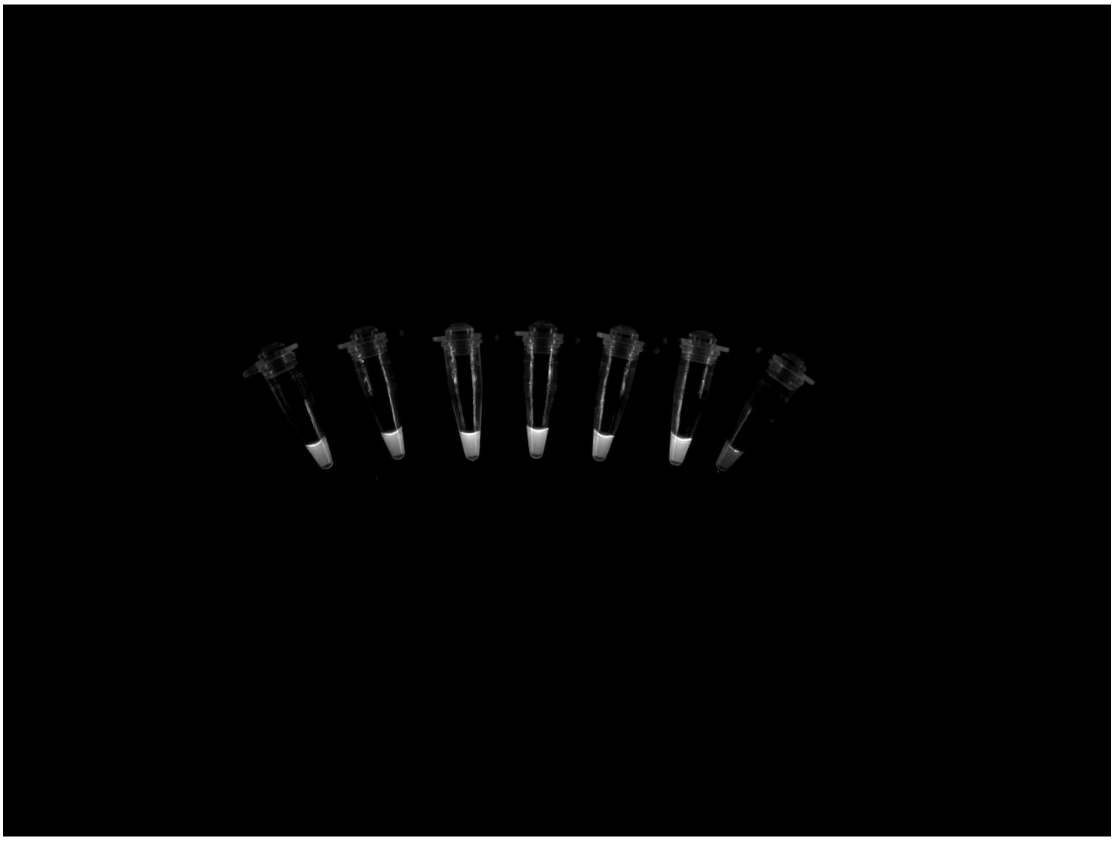

Figure 5B was generated from that original image.

Direct observation by naked eye when the Cas12a reactions system exposed to blue light. The detected nucleic acid samples were obtained by RPA at varied time from 5 to 30 minutes. From the left to the right concentration respectively is 30 minutes, 25 minutes, 20 minutes, 15 minutes, 10 minutes, 5 minutes.
